# Fracture Behavior and Mechanism of Nb-Si-Based Alloys with Heterogeneous Layered Structure

**DOI:** 10.3390/ma17112735

**Published:** 2024-06-04

**Authors:** Sheng Wang, Xiaoli Wang, Zhiming Wang, Zhiping Sun, Weicheng Ye, Qihu Zhao

**Affiliations:** 1 School of Mechanical Engineering, Qilu University of Technology (Shandong Academy of Sciences), Jinan 250353, China; 10431210043@stu.qlu.edu.cn (S.W.); zhi820426@163.com (Z.W.); benzsun@163.com (Z.S.); 19854199505@163.com (W.Y.); 18631669546@163.com (Q.Z.); 2 Shandong Institute of Mechanical Design and Research, Jinan 250031, China

**Keywords:** Nb-Si-based alloys, heterogeneous layered structure, fracture behavior, mechanical alloying, SPS

## Abstract

Novel Nb-Si-based alloys with heterogeneous layers that have the same composition (Nb-16 at.%Si) but different phase morphologies were designed in this work. Heterogeneous layered structure (HLS) was successfully fabricated in Nb-16Si alloys by layering composite powders after various degrees of mechanical alloying (6 h, 12 h, 18 h, and 24 h) alternately and subsequent spark plasma sintering (SPS). The influence of HLS on the fracture behavior at both room and elevated temperature was investigated via single-edge notched bending (SENB) and high-temperature compression, respectively. The results show that the diversified HLS is obtained by combining hard layers containing fine equiaxed crystals and/or soft ones with coarse lamellar niobium solid solution (Nbss). By affecting the crack propagation in SENB, HLS is favorable for improving the fracture toughness and exhibits a significant increase compared with the corresponding homogenous microstructure. Moreover, for the same HLS, a more excellent performance is achieved when the initial crack is located in the soft layer and extended across the interface to the hard one through crack bridging, crack deflection, crack branching, and shielding effect. Fracture starts in the soft layer (from powders of ball-milled for 12 h) of a 12–24 alloy, and a maximum *K_Q_* value (14.89 MPa·mm^1/2^) is consequently obtained, which is 33.8% higher than that of the homogeneous Nb-16Si alloy. Furthermore, the heterogeneous layered alloys display superior high-temperature compression strength, which is attributable to the dislocation multiplication and fine-grained structure. The proposed strategy in this study offers a promising route for fabricating Nb-Si-based alloys with optimized and improved mechanical properties to meet practical applications.

## 1. Introduction

Refractory Nb-Si-based alloys have a high melting point, low density, excellent elevated-temperature strength, and creep resistance and are perceived as critical substitute high-temperature structural material above 1150 °C for existing nickel-based high-temperature alloys in the aerospace field [1,2,3]. Ductile niobium solid solution (Nbss) and strengthening niobium silicides (Nb_5_Si_3_ and/or Nb_3_Si) comprise the multiphase complex structure of Nb-Si binary alloys [4,5,6]. However, the low room-temperature fracture toughness resulting from the intrinsic brittleness of intermetallic compounds is the nonnegligible bottleneck of their practical application [7,8,9]. In order to optimize the mechanical properties of Nb-Si-based alloys, researchers have made great efforts to explore novel alloying strategies and refining production [10,11]. Up to now, many different alloying elements have been added for an excellent balance of strength and toughness. Ti hinders crack extension by lowering the Peierls–Nabarro energy barrier [12], while Hf and Mo increase the volume fraction of Nbss to improve fracture toughness [13,14]. However, as the number of alloying elements increases, it becomes difficult to accurately elucidate the correlation between the elemental composition and mechanical properties [15]. Furthermore, most elements have an unfavorable effect on the high-temperature performance due to the lower melting point than Nb [16]. For the preparation process, hot extrusion combined with heat treatment results in the decomposition of metastable Nb_3_Si and an increased amount of Nbss, which is favorable to fracture toughness at the expense of reducing its strength [17]. An analysis of the research mentioned above shows that the existing works focus on optimizing the homogeneous microstructure of Nb-Si-based alloys to enhance the fracture toughness, yet it usually severely damages other properties. There is still no effective way to counteract the intrinsic brittleness of Nb-Si-based alloys without seriously compromising the necessary performance.

Recently, designing heterogeneous structure (HS) has been regarded as a promising approach to achieving a balance between strength and toughness [18,19]. HS has been reported to effectively enhance strength and ductility, which is attributed to the coupling of multiple deformation behaviors and mechanisms caused by mechanical incompatibility between soft and hard structures [20,21,22]. Nature’s nacreous layer, combining an exceptionally tough matrix with a high-strength one, has inspired the development of heterogeneous layered structure (HLS) materials to optimize the strength and toughness [23]. HLS, as a characteristic type of HS structure, is used to improve the inherent brittleness of intermetallics [24]. It has been found that the bimaterial interface of hetero-junction markedly diminishes the fatigue crack extension rate when the crack starts from the soft layer to the hard one [25]. However, the applicability of this mechanism to Nb-Si alloys has not been investigated so far. Powder metallurgy technology has been adopted to fabricate HLS materials, achieving the superior synergy of strength and ductility [26]. Most of the current studies tend to laminate two different substances to prepare HLS materials. Du [27] used powder metallurgy to fabricate Ti-N alloys with laminated structures. The unique strengthening mechanism in layered Ti-N leads to high-stress accumulation and induces the sprouting of micropores to produce the toughening mechanism of crack deflection. So far, there are very few reports about the preparation of Nb-Si alloys with HLS using the powder metallurgy process, except for when the HLS alloy was prepared using SPS by stacking separate Nb with Nb_5_Si_3_ powders alternately [28]. However, the result was not satisfactory because of the neglected brittle/ductile phase ratio and interfacial mismatch between Nb and Nb_5_Si_3_ [29]. This study is expected to make an improvement through adjustment to the volume fraction and morphology of Nbss and silicides with the fixed atomic ratio of Nb and Si by mechanical alloying and SPS. In addition, based on the research gap of the influence of crack sources on the fracture behavior of Nb-Si alloys, the notch was designed to be located at the soft/hard layer in the SENB test of HLS alloys.

In this work, Nb-16Si (at.%) alloys with HLS were successfully prepared using mechanical alloying and subsequent SPS, and then the microstructures were explored. The influence of HLS on the fracture behavior at both room and elevated temperatures was investigated via SENB and high-temperature compression, respectively. Furthermore, the mechanism of the crack source effect on fracture toughness of Nb-16Si alloys with HLS was elaborated. Based on previous studies, this work is expected to improve the fracture toughness of Nb-Si-based alloys without significantly decreasing their strength. Therefore, it provides a promising way to optimize the properties of Nb-Si-based alloys and meet specific material requirements and further application.

## 2. Experimental Procedures

**Figure 1:**
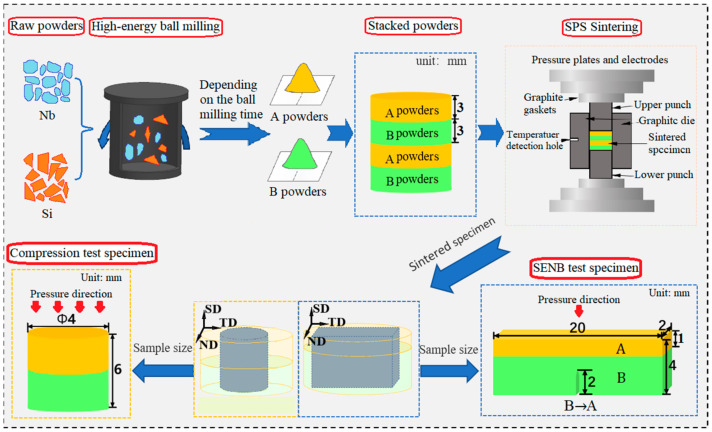
Schematic diagram of specimen preparation procedure and dimensions for mechanical property testing. The coordinate axes are represented by the sintering direction (SD), normal direction (ND), and tangential direction (TD), among which the loading direction is parallel to SD.

Figure 1 delineates the preparation procedure and dimensions of the test specimens. ‘A powders’ and ‘B powders’ represent the powders ball-milled for Ah and Bh, respectively.

### 2.1. Materials Preparation

**Figure 2:**
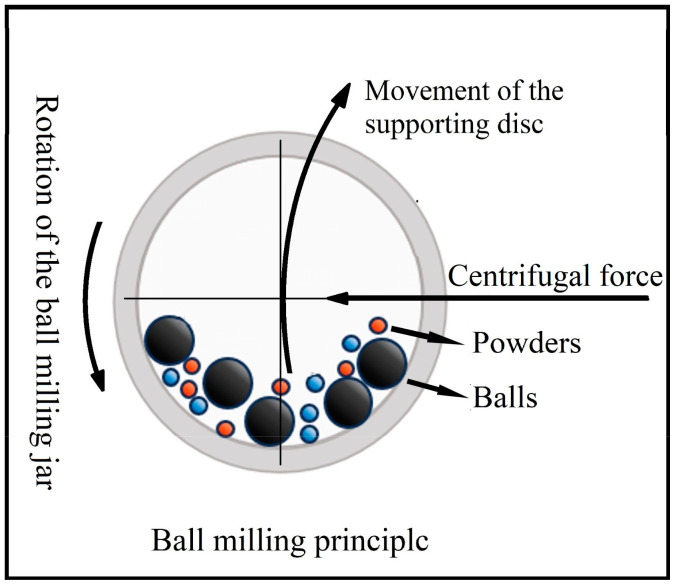
Ball milling principle of schematic diagram.

Commercial Nb (purity 99.9%, particle size 75 µm) and Si (purity 99.6%, particle size 75 µm) element powders were used as the raw materials to prepare Nb-Si-based alloys in this study. A mixture of metal powders with a nominal composition of 84 at.% Nb and 16 at.% Si was subjected to high-energy ball milling in a planetary ball-milling machine (QM-BP) with stainless steel vacuum ball milling jars (250 mL) and GCr15 bearing steel balls (10 mm in diameter). Figure 2 illustrates the ball-milling machine and principle. In order to improve the efficiency of ball milling, high-energy ball milling was carried out at room temperature using a ball-powder mass ratio of 12:1 and a rotation speed of 300 rpm. Anhydrous ethanol was added as the process control agent to reduce agglomeration during ball milling. Composite powders were continuously milled for 6, 12, 18, and 24 h to investigate the phase formation process.

In order to obtain the HLS in Nb-16Si binary alloys, the above ball milled powders milled various times were packed into a graphite mold (30 mm of internal diameter, 70 mm of external diameter, 70 mm of height) layer by layer alternately with the match of 6–18 h, 6–24 h, 12–18 h, and 12–24 h. They were subsequently consolidated through SPS at 1450 °C under a uniaxial pressure of 30 MPa for 10 min in a dual-power spark plasma sintering furnace, SPS-625HF (Fuji Electronic Industrial Co., Ltd., Sagamihara, Japan). The heating and cooling rates are 150 and 50 °C/min, respectively, with a vacuum level of 5 Pa during the sintering process. The corresponding sintered bulks, with each layer having 3 mm in thickness, were named 6–18, 6–24, 12–18, and 12–24, respectively. Nb-16Si alloys with homogeneous microstructure were also fabricated from separate high-energy ball-milling powders following the same sintering process for comparison.

### 2.2. Microstructural Observation

The phase composition of both composite powders from mechanical alloying and sintered blocks was characterized by X-ray diffraction (XRD, Rigaku SmartLab, Tokyo, Japan) with Cu Kα1 radiation (λ = 1.54056 Å) at an angle range of 20–80° with steps of 0.02° at room temperature. The changes in particle morphology during the milling and microstructure of the sintered specimens were characterized using a scanning electron microscope (SEM, Phenom Prox, Eindhoven, The Netherlands) with backscattered electron (BSE) imaging. The specimens for SEM observation were ground with metallographic abrasive paper and then mechanically polished with 2.5 μm diamond paste. The composite powders were embedded into the phenolic molding compound with the metallographic mounting machine. Then, the cross-section of particles was sandpapered to observe the microstructure. The chemical homogeneity was checked with X-ray microanalysis using an EDS spectrometer (Oxford Instruments, Abingdon, UK, INCA 200 soft). Electron backscatter diffraction (EBSD, EDAX Hikari Plus, Silicon Valley, CA, USA) was applied to investigate the deformation of microstructures after high-temperature compression. The EBSD samples were first inlaid in a phenolic molding compound. After that, they were mechanically polished in the same way as the SEM ones and then vibrationally polished with a 0.05 μm colloidal silica suspension for 8 h.

### 2.3. Mechanical Property Tests

**Figure 3:**
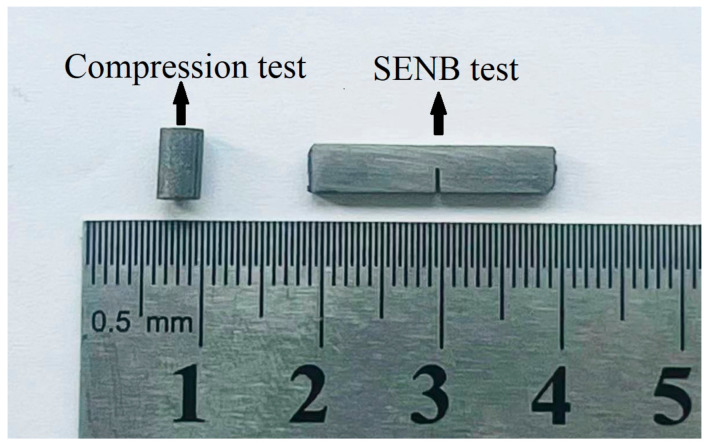
High-temperature compression and SENB specimens.

Specimens for mechanical property tests were prepared using wire electrical discharge machining (WEDM). The specimens prepared using WEDM (Figure 3) were ground on abrasive papers to remove the heat-affected surface. Vickers hardness was measured with an HV-1000A digital hardness tester under a load of 9.8 N with a dwell time of 15 s. The average value was obtained from at least five indents on each tested object at 20 µm intervals from the interface to both sides. Facture toughness was evaluated using single-edge notch bending (SENB) at a displacement rate of 0.05 mm/min at room temperature on the AGS-X5KN universal testing machine. Cross-layer specimens were cut into 20 mm length (*S* = 16 mm), 4 mm width, and 2 mm thickness. A straight notch of about 2 mm depth was inserted into different heterogeneous layers at the midpoint using WEDM with a 0.1 mm diameter wire. Each test was repeated thrice to obtain the average for accuracy. The room temperature fracture toughness can be expressed by *K_Q_*, and the *K_Q_* was calculated using the following Equations (1) and (2) [30]:

(1) $$K_{Q}=\left(\frac{P\cdot S}{B\cdot W^{3/2}}\right)\cdot f\left(\frac{a}{w}\right)$$

(2) $$f\left(a/w\right)=3{\left(\frac{a}{w}\right)}^{\frac{1}{2}}\cdot \frac{1.99-(\frac{a}{w})(1-\frac{a}{w})\left[2.15-3.93\left(\frac{a}{w}\right)+2.7{\left(\frac{a}{w}\right)}^{2}\right]}{2(1+2\frac{a}{w}){\left(1-\frac{a}{w}\right)}^{\frac{3}{2}}}$$

*P* is the maximum force during testing (KN). *a* denotes the length of the inner groove housing the pre-existing crack. *B* is the specimen thickness (cm). *W* signifies its width (cm). *S* is the distance between two pivot points of the experiment (cm), given by *S* = 4*W*.

The high-temperature compression test was, respectively, performed at 1050 °C, 1150 °C, and 1250 °C under the same strain rate of 0.01 S^−1^ using the Gleeble 3500C thermal simulation tester. Cylindrical cross-layer specimens with 6 mm in height and 2 mm in diameter were applied. The loading direction was parallel to the sintering direction and perpendicular to heterogeneous layers in both experiments of SENB and high-temperature compression.

## 3. Results and Discussion

### 3.1. Microstructure Evolution of Ball-Milled Powders

**Figure 4:**
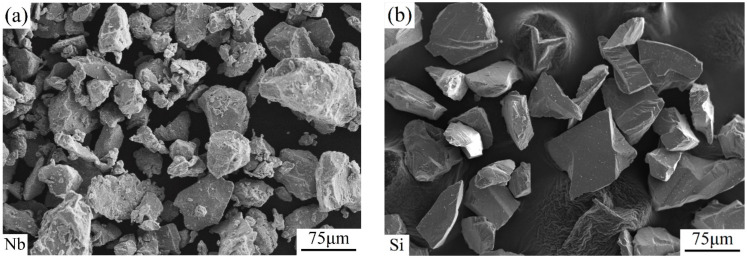
Morphologies of raw elemental powders of Nb (**a**) and Si (**b**).

**Figure 5:**
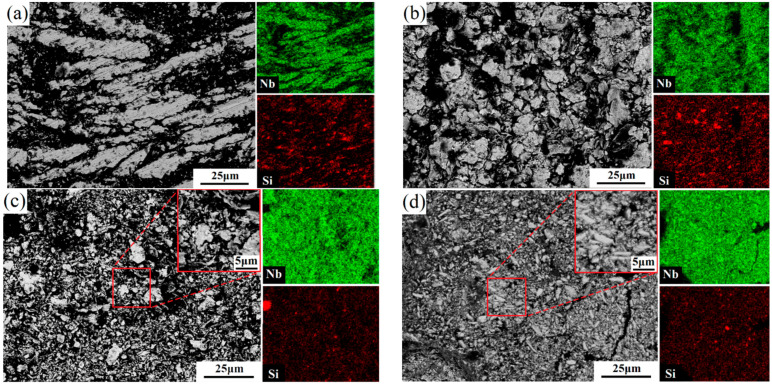
Cross-section microstructures and elements distribution of ball-milled Nb-16Si composite powders for 6 h (**a**), 12 h (**b**), 18 h (**c**), and 24 h (**d**).

The morphologies of raw elemental powders (Nb and Si) are presented in Figure 4. They have an irregular shape and a particle size of about 75 μm. The cross-sectional microscopic morphology evolution and distribution of elements of Nb-16Si composite powders during milling are illustrated in Figure 5. In the beginning stages of high-energy ball milling, ductile Nb particle shape varied from irregular polygon to large slice due to severe plastic deformation, while brittle Si powders were immediately collided into small size particles by the grinding balls. Composite powders milled for 6 h have characteristically thick slices of a broad range of sizes that were assigned to the ductility of Nb. For 12 h milled powders, the particle size greatly reduced to about 10 μm, while the morphology was still coarse and flaky. The non-uniform elemental distribution was observed from the EDS mapping. In the addition of anhydrous ethanol, the flesh interfaces of milled powders were separated, so the cold-welding process and agglomeration were suppressed. With the duration increasing, flaky Nb particles became fragile because of work hardening under subsequent pressing and shearing. The particle size was continuously reduced during the milling process, which was more noticeable after 18 h of milling. With milling time further increasing to 24 h, powders consisted of fine and flocculent particles, and the chemical composition became uniform.

**Figure 6:**
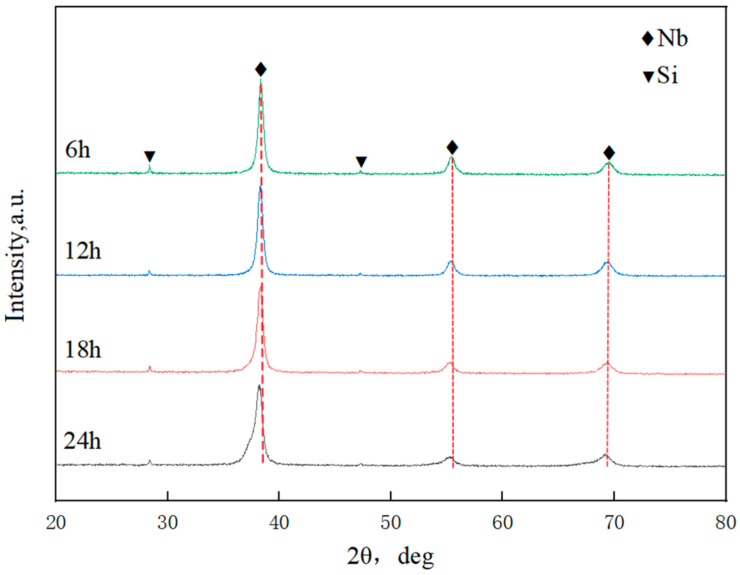
XRD patterns of Nb-16Si composite powders milled at different times.

Figure 6 shows the XRD patterns of Nb-16Si composite powders after different milling times. Nb and Si characteristic peaks were confirmed. The intensity of Si peaks was weak, possibly because the amount of Si was small, and a substantial portion of Si particles were embedded into Nb powders. No significant change was detected after milling for 6 h and 12 h. Nevertheless, Nb peaks of 24 h milled powders clearly weakened and broadened. It indicated a decrease in the grain size and an increase in the microstress and detection of deformed Nb particles. Moreover, severe plastic deformation resulted in distorting lattice and lattice expansion, and thus, the Nb diffraction peaks significantly shifted to a small angle. In addition, no definite proof for the formation of niobium silicides (Nb_5_Si_3_ or Nb_3_Si) was found.

### 3.2. Microstructural Characterization of Heterogeneous Nb-16Si Alloys

**Figure 7:**
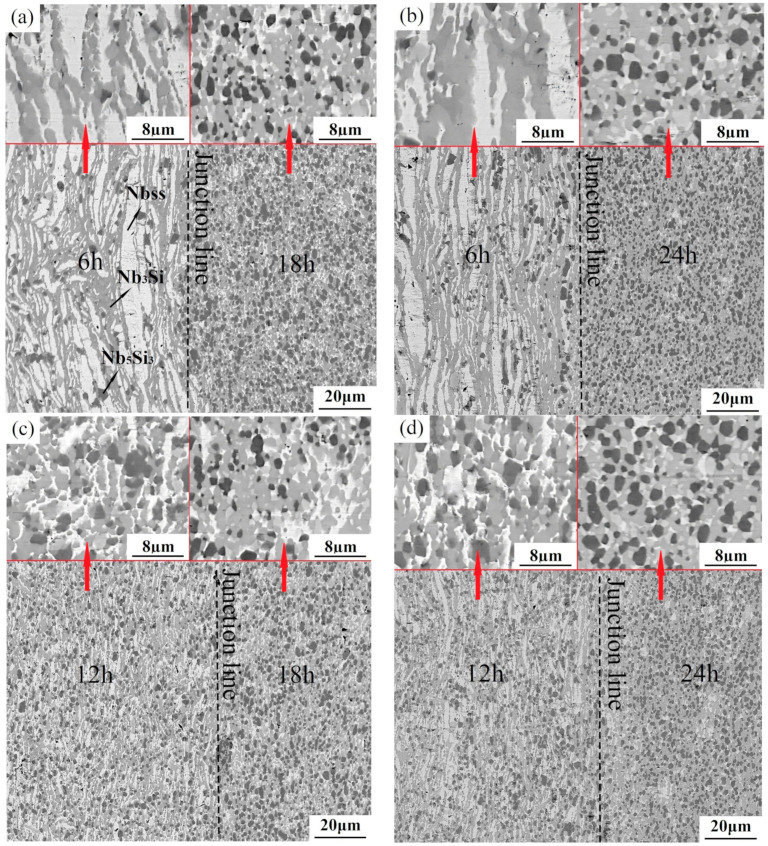
BSE images of Nb-16Si alloys with HLS: 6–18 (**a**); 6–24 (**b**); 12–18 (**c**); 12–24 (**d**).

Figure 7 presents the BSE images of Nb-16Si alloys with HLS obtained by SPS from various combinations of two ball-milled powders, as mentioned earlier, including 6–18 h, 6–24 h, 12–18 h, and 18–24 h. Consequently, the sintered alloys were marked as 6–18, 6–24, 12–18, and 12–24, respectively. Local enlarged images at the top exhibit the details of each individual layer from the specifically single kind of composite Nb-16Si powders. The interfaces did not align along a straight line. Instead, microstructures on both sides joined together in union without a clear boundary between layers, which improved the interface bonding performance. It resulted from the inevitable overlap and misalignment when loading powders into the graphite mold.

**Figure 8:**
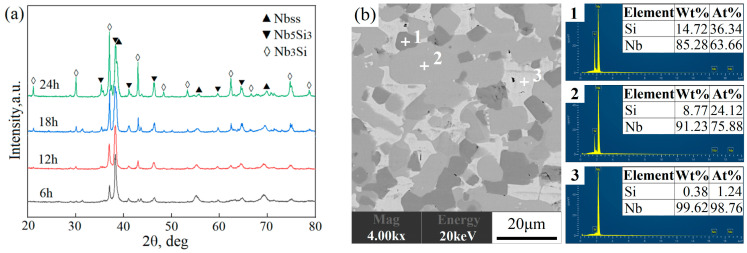
XRD pattern (**a**) and EDS analysis (**b**) of the sintered Nb-16Si alloy.

Results from the XRD pattern and EDS analysis (Figure 8) of the sintered Nb-16Si alloy in conjunction with the literature [31] show that the material consisted of white Nbss, gray Nb_3_Si, and dark-gray Nb_5_Si_3_.

Large lamellar Nbss phases with the highest concentration were surrounded by intermetallic compounds in the layer from 6 h milled powders (labeled as 6 h layer; other layers and alloys were labeled in the same manner) exhibiting a typical heterogeneous structure. Furthermore, besides the above three phases, a small number of pores were also found in black. It probably resulted from the low surface activities of the powder in the initial stage of ball milling, which harmed sintering densification [32]. Although the content of the solid solution declined, thinner and smaller Nbss flakes were still discernible up to 12 h. With the increment of milling duration to 24 h, fewer flake-like crystals were observed, the coarse grains transformed into much finer equiaxed ones (about 3 μm in diameter), and a more uniform microstructure was obtained in separate layers. Moreover, the bulk volume fraction of both Nb_3_Si and Nb_5_Si_3_ markedly increased, and pores were almost invisible. It implied that the combination action and reactivity activation on composite powders after prolonged milling time promoted in situ reaction and densification when sintering. The microstructural diversity mentioned above verified the feasibility of designing and realizing the heterogeneous layers in Nb-Si-based alloys (Figure 7) using a flexible combination mode.

### 3.3. Room-Temperature Fracture Behavior of Heterogeneous Nb-16Si Alloys

**Figure 9:**
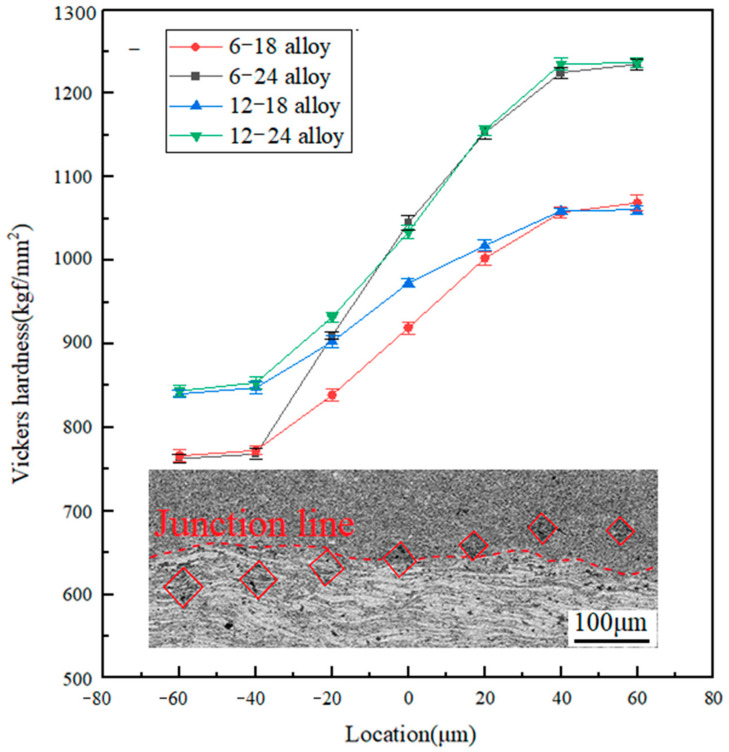
Vickers hardness and hardness point distribution of Nb-16Si alloys with HLS. Zero and negative and positive numbers on the horizontal axis represent the areas of interface short and long milling times, respectively.

The Vickers hardness distribution from the interface to both sides of Nb-16Si alloys with HLS is displayed in Figure 9. Mean absolute error (MAE) was chosen to count the potential errors [33], and the error rate was calculated to be 0.68%, indicating a high accuracy. The hardness of all the sintered alloys showed similar rising trends from short ball-milling times to long ones. It indicated an excellent interface bonding formed between two different powders during the sintering process. It was also detected through morphology observations of closely combined junctions between two layers of materials (Figure 7). In addition, the 24 h layer possessed the most outstanding hardness because it had the maximum volume fraction of niobium silicides with fine equiaxial grains. The hardness of the 6 h layer, on the other hand, was the lowest due to the amount of soft Nbss in coarse and irregular shapes. Overall, the hardness of the layers decreased as the milled time became longer.

**Figure 10:**
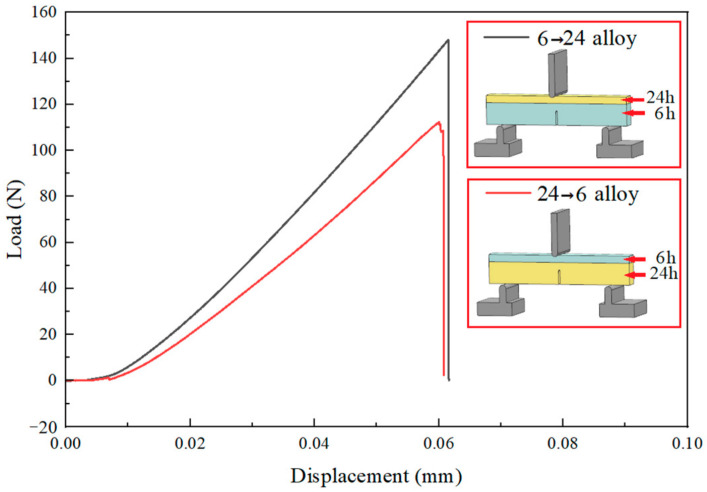
Load-displacement curves of SENB test of heterogeneous 6–24 alloy.

To investigate the influence of crack initiation site, propagation direction and interface on the fracture behavior and toughness, the notch was cut into different layers of HLS during SENB testing. Figure 10 displays the true load-displacement curves of SENB and a schematic diagram of cross-layer specimens with opposite crack origins of heterogeneous 6–24 alloys. 6→24 symbolizes that the crack started from the 6 h layer and then expanded across the junction to 24 h one. 24→6 indicates the opposing meaning. By comparing these two tests, 6→24 had the better ability to resist crack growth at the same deformation.

**Figure 11:**
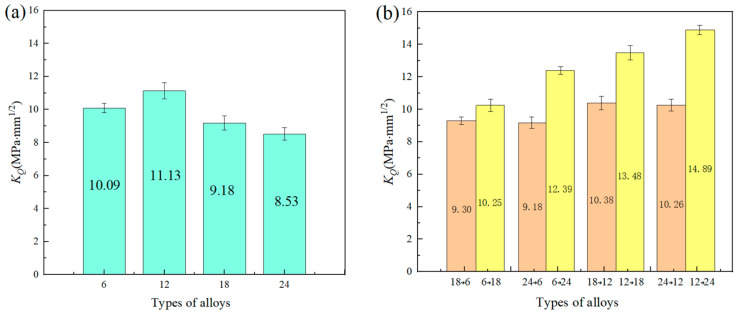
*K_Q_* values of Nb-16Si alloys with homogeneous (**a**) and heterogeneous layered (**b**) microstructure.

**Figure 12:**
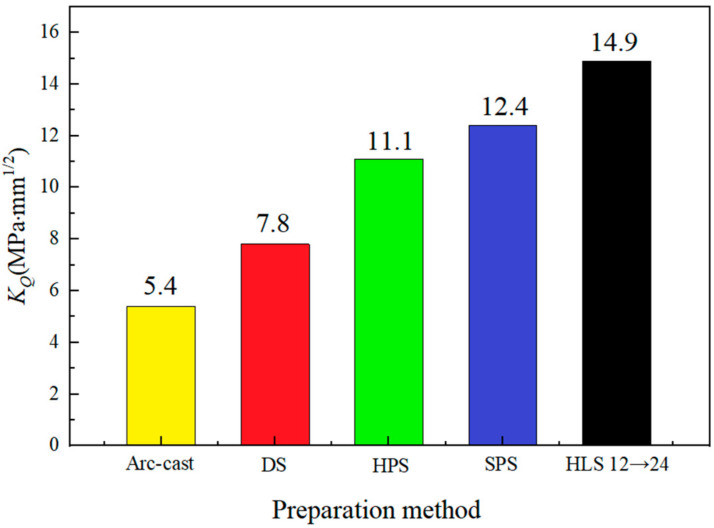
Room-temperature fracture toughness of Nb-16Si alloys fabricated using different preparation methods.

The bending test under the same condition as the above was performed on all the other studied Nb-16Si alloys with HLS. For comparison, the fracture toughness of a single homogeneous material was also measured. The corresponding *K_Q_* values are illustrated in Figure 11. MAE was used to calculate potential errors, and the error rate was 3.39%. For the homogeneous material system, the result exhibited a non-linear relationship as the ball-milling time increased. Though the 6 h alloy had the highest content and the largest size of Nbss (Figure 7), which was beneficial for increasing the fracture toughness, the sparse pores were not neglected in cracking failure. Therefore, 12 h alloy unexpectedly reached a peak, owning a considerable volume of Nbss with finer grain size and high density. The 18 h and 24 h alloys successively decreased with the prolonging of ball-milling time because of an increase in the amount of niobium silicides. Nevertheless, the result of Nb-16Si alloys with HLS seemed more complicated. Most noteworthy, HLS improved the fracture toughness and displayed a significant increase compared with the corresponding homogenous microstructure. Moreover, for the same HLS, more excellent performance was brought out when the initial crack was located in the soft layer and extended across the interface to the hard one, and 12→24 reached a maximum *K_Q_* value (14.89 MPa·mm^1/2^). It was 16.8% higher than the best one of the Nb-16Si alloys obtained with arc-cast, directional solidification (DS), hot-pressed sintering (HPS), and SPS (Figure 12) [34,35]. The detailed fracture behavior and toughening mechanism were elaborated based on the following metallographic observation and analysis of fracture morphology and crack propagation path.

**Figure 13:**
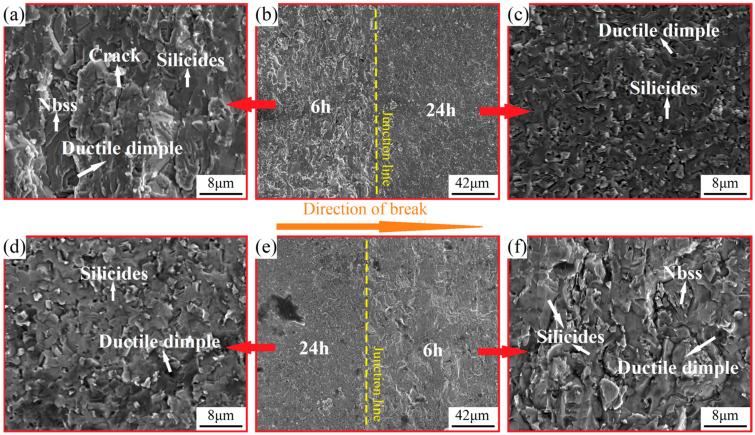
Fracture morphology of 6→24 (**a**–**c**) and 24→6 (**d**–**f**) alloys (6 h layer (**a**), junction (**b**), and 24 h layer (**c**) of 6→24 alloy; 24 h layer (**d**), junction (**e**), and 6 h layer (**f**) of 24→6 alloy).

Scanning electron microscopy analysis of heterogeneous layered Nb-16Si alloys was performed by observing the microscopic appearances of the fracture surface and cross-section. The 6–24 alloy consisting of the greatest two layers was investigated first. Figure 13 illustrates the bending SEM fractographs of both the 6→24 and 24→6 alloys. The fracture traversed the matrix, as well as the interface. It revealed the exceptional interface layer of metallurgical bonding and different fracture modes between 6 h and 24 h layers. At lower magnification (Figure 13b,e), the fractography of 24→6 alloy exhibited a much smoother surface, indicating a weaker fracture resistance [36]. Such a finding was consistent with the results from the bending tests mentioned above. According to the morphology at higher magnification, the fracture of heterogeneous multiphase Nb-16Si alloys was a mixed ductile-brittle fracture, not simply a typical brittle or ductile fracture. Nb solid solution was characterized by ductile rupture with plastic-tearing features and/or cleavage fraction with river pattern, which was determined based on the orientation characteristic of slip planes. The smooth appearance of brittle silicides suggested the fracture a mode of transgranular-type fracture. Therefore, the surface of fracture in the 6 h layer (Figure 13a,f) was humpier and hillocky owing to the ductile fracture behavior of the higher proportion of coarser Nbss. The mixed mode fracture was more pronounced in 6→24 alloy. Such behavior of fracture contributed to the energy dissipating, thereby improving the fracture toughness [37].

**Figure 14:**
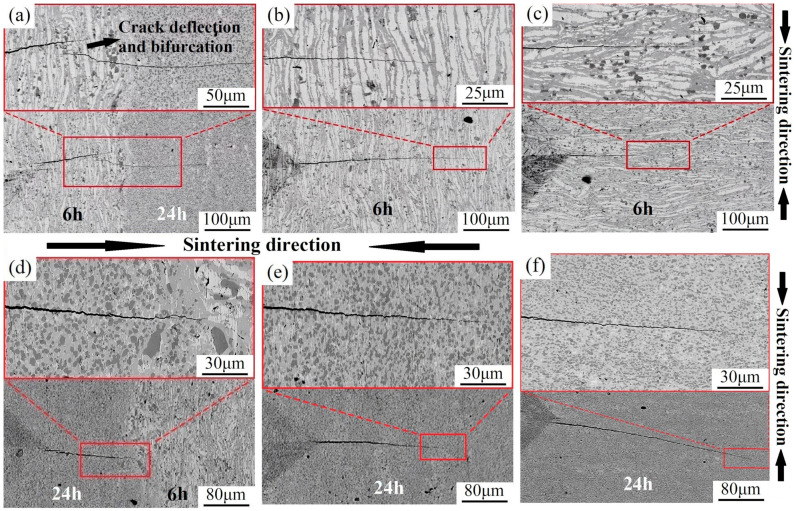
Crack propagation of the 6–24 alloy in the indentation test (6→24 (**a**), 6 h layer (**b**,**c**), 24→6 (**d**), 24 h layer (**e**,**f**), interface layer (**a**,**d**), in the direction parallel (**b**,**e**), and perpendicular (**c**,**f**) to sintering direction).

Crack extension direction in the indentation test was employed to elucidate the effects of HLS, interface layer, Nbss, and niobium silicides on the fracture behavior and mechanism of Nb-16Si alloys. Figure 14 provides the details of crack propagation paths in the 6–24 alloy. In 6→24 (Figure 14a), it could be observed that the interface blocked crack growth and changed the propagation direction of the main crack when the initial crack was located in the soft zone. It resulted from the higher crack driving force in the harder layer. The Nb-16Si alloys with HLS in this study were characterized by heterogeneous microstructure with different volume fractions, morphology, and grain size in each layer. In HLS, the crack initiation, propagation, and fracture are generally correlated with the crack driving force (a configuration force). When a crack propagated from the soft layer to the hard one in the 6→24 alloy, the fracture driving force diminished and decreased the crack propagating rate and even prevented cracks from further propagation. Such a phenomenon was termed as the shielding effect [38,39]. Conversely, the anti-shielding effect with positive *C_inh_* enhanced the crack driving force and accelerated its expansion in the 24→6 alloy.

In addition, in the 6→24 alloy, crack deflection and bifurcation subsequently occurred close to the interface, which absorbed more fracture work and greatly increased crack tolerance. When a crack extended near the hard layer from the soft one, interfacial tensile stress was induced and worked as a repulsive force, leading the crack to deflect or bifurcate [40,41]. The branch crack became far narrower after that, which demonstrated that these processes contributed to major energy dissipation, decreased the driving force for crack propagation, and thereby enhanced the fracture toughness of the 6→24 alloy remarkably. However, in the 24→6 alloy, the interfacial tensile stress as an attractive force led the crack to traverse the joint without deviation, which conserved energy and reduced the toughness.

Moreover, crack deflexion also took place at phase and grain boundaries, which was more significant in the 24 h layer with fine grains (Figure 14e,f). In addition, when the crack propagated in a direction parallel to the sintering direction in the 6 h layer (Figure 14b), the crack traversed coarse elongated Nbss and deflected more frequently than in a direction perpendicular to the sintering direction (Figure 14c). It was because of the presence of more phase boundaries of solid solution and intermetallics along the crack growth path in the former. Moreover, crack bridging occurred at some white solid solution regions, and ductile phase Nbss released the energy by undergoing plastic deformation. Consequently, the stress intensity at the crack tip decreased, which inhibited crack extension and enhanced the toughness.

**Figure 15:**
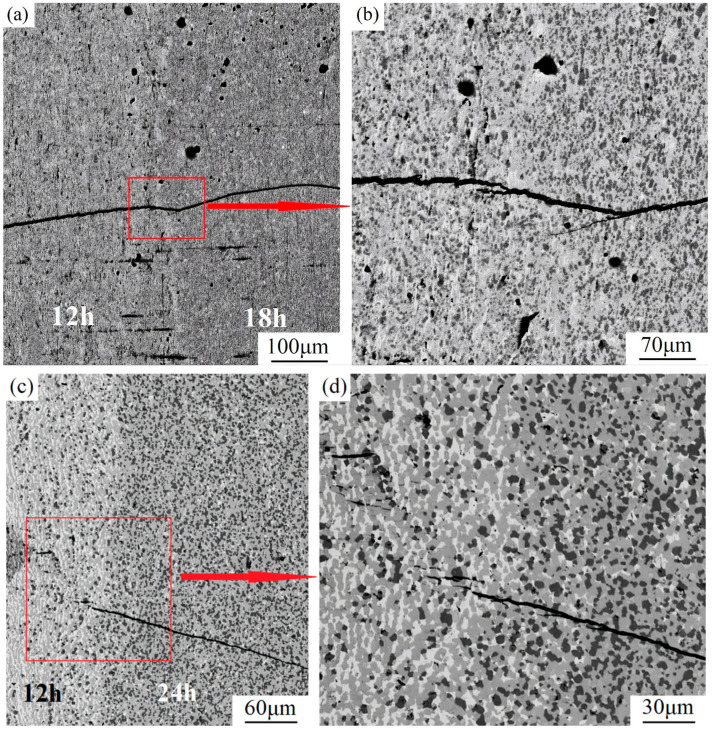
Crack growth paths crossing the interface of 12→18 (**a**,**b**) and 12→24 (**c**,**d**) in the indentation test.

Based on the above analysis, indentation tests were also performed on the 12→18 and 12→24 alloys to analyze the crack extension and fracture behavior of Nb-16Si with HLS further. Corresponding results are displayed in Figure 15. Crack deflection and bifurcation likewise took place at the interface when the crack started in the soft layer and extended to the hard matrix. Crack furcation dissipated some of the energy, restrained the main crack growth and diminished the crack tip stress intensity factor. Remarkably, in the 12→24 alloy, the sharp corner of the indentation in the softer layer was not the cracking point of the main crack, which appeared initially at the interface and spread forward in the 24 h layer. Even more dramatically, before that, some scattered little cracks began from indentation and changed crack propagating orientation. The cracking behaviors described above were conducive to obtaining optimizing toughness.

### 3.4. High-Temperature Fracture Behavior of Heterogeneous Nb-16Si Alloys

**Figure 16:**
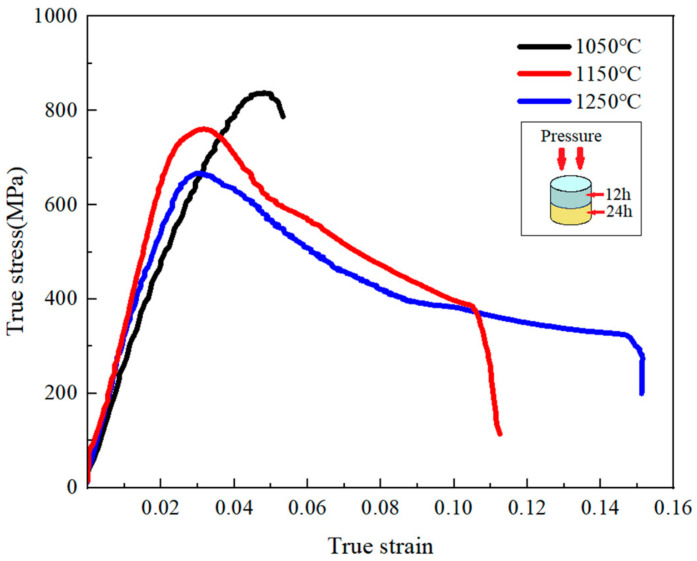
Compressive true stress–strain curves of the heterogeneous layered 12–24 alloy at different temperatures.

**Figure 17:**
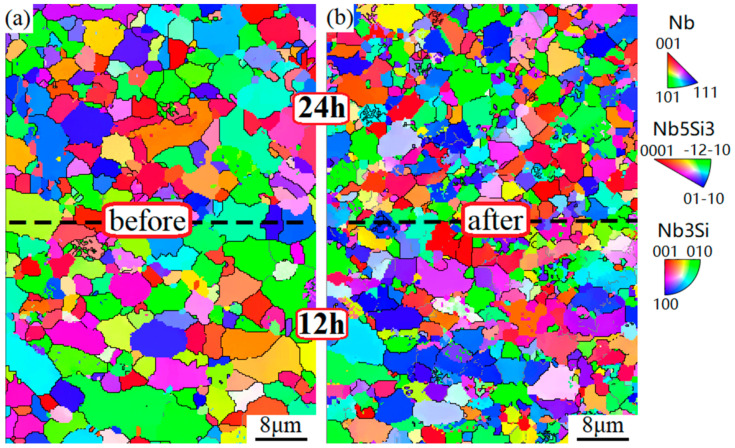
EBSD IPF maps of the 12–24 alloy before (**a**) and after (**b**) compression at 1250 °C.

The high-temperature compressive true stress–strain curves of heterogeneous layered 12–24 alloy with the highest fracture toughness at different temperatures are presented in Figure 16. The results revealed the absence of definite yields in all the alloys. During the initial deformation, the flow stress of each alloy was acutely enhanced with increasing deformation degree due to the effect of work hardening, and the rise developed slowly because of dynamic recovery. However, compressive strength decreased with increased temperature, and the maximum peak was reached at 1050 °C. In addition, after the peak point, different deformation and mechanical behaviors were detected at different temperatures. The alloys cracked as they reached the peak flow stress and exhibited brittle fractures without dynamic recrystallization at the lower 1050 °C. Nevertheless, at 1150 °C and 1250 °C, the flow stress dropped rapidly within the strain range from 0.03 to 0.04. EBSD analysis was conducted on the 12–24 alloy before and after high-temperature compression at 1250 °C. The Inverse Pole Figure (IPF) maps are shown in Figure 17. Observation of the grain map reveals that high-temperature compression produces a large number of ultrafine grains because of the considerable amount of grain boundaries from the superfine structure, which was in favor of the recrystallization nucleation and softening process [42]. With the development of recrystallization, the flow stress of the latter deformation remained essentially unchanged when a balance between hardening and softening was achieved. The follow-up descent part became gentle and enjoyed similar features. Overall, it was very gratifying that the heterogeneous layered 12–24 alloy demonstrated an outstanding compressive strength (over 600 Mpa) without reducing its ductility in comparison to the alloys with ultrafine grains [43].

**Figure 18:**
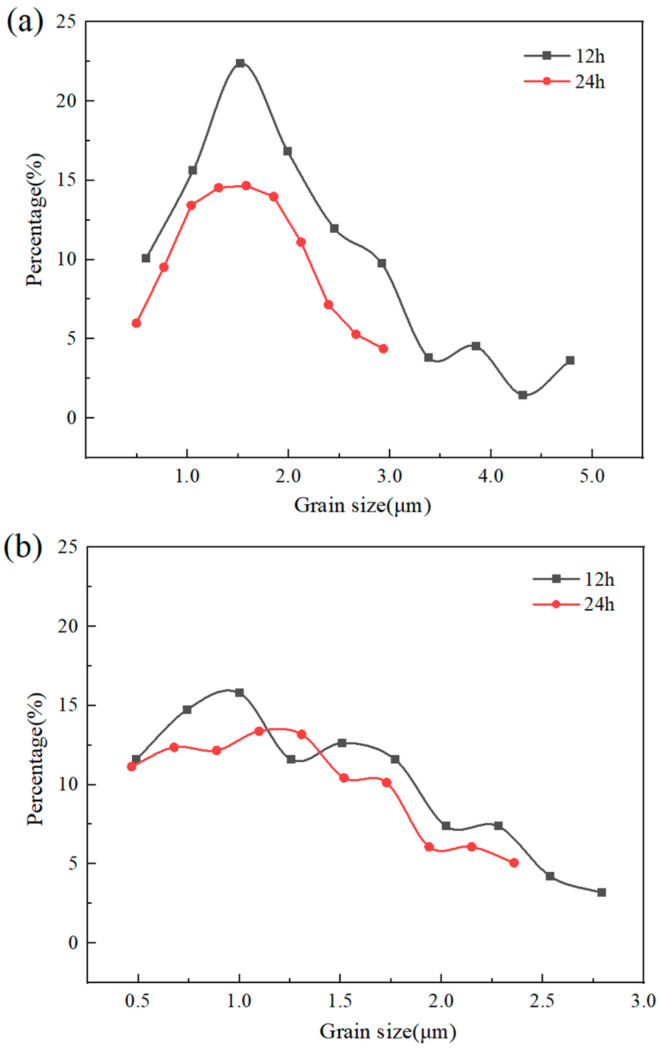
Grain size distribution of each layer of the 12–24 alloy before (**a**) and after (**b**) compression at 1250 °C.

The crystallite size distribution obtained by analyzing the above IPF maps is shown in Figure 18. It displayed that the grain size of the 12 h layer invariably remained bigger than that of the 24 h one, and their mean grain sizes were approximately 2.35 μm and 1.73 μm (Figure 18a), respectively. However, subjected to compression deformation at high temperatures, both layers decreased the crystal size and had uniform grain size. The difference between before and after compression diminished later, resulting from dynamic recrystallize refinement, and their mean grain sizes reduced to 1.5 μm and 1.3 μm, respectively (Figure 18b).

The results from the IPF maps (Figure 17) further demonstrated that a dynamic recrystallization leads to the efficient refinement effect. Consequently, both layers had fine isometric crystal structures, and the preferred orientation detected in the 12 h layer also disappeared after deformation. The fine-grained structure was in agreement with the grain size distribution above (Figure 18). Under pressure stress, plastic deformation of the ductile Nbss phase resulted in dislocation multiplication and tangle. Brittle silicides fragmented into small pieces and increased the grain boundary energy. These two factors were favorable for nucleation and a driving force of recrystallization when the critical deformation was exceeded.

**Figure 19:**
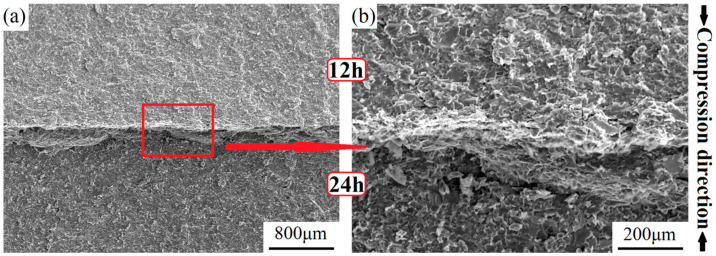
Fracture morphology of the heterogeneous layered 12–24 alloy at low (**a**) and high magnification (**b**) after compressive deformation at 1250 °C.

Cross-section fractographies of the heterogeneous layered 12–24 alloy compressed at 1250 °C are outlined in Figure 19. Remarkably, a raised ridge was positioned along the interface where the fractured surface intersected from opposite directions after undergoing different fracture behaviors in both layers. According to the observed morphology in high magnification, it was featured with a mixed ductile–brittle fracture presenting certain dimples and plastic-tearing features by tensile stress. The concave blow indicated that a similar ridge structure existed on the other half of the fracture specimen. The fracture surface of the 12 h and 24 h layers both displayed a quasi-cleavage fracture being characterized by a quasi-cleavage plane. The 12 h layer owned particularly slightly larger flat surfaces and tearing edges because of the marginally coarser multiphase microscopic structure. It is speculated that Niobium silicides exhibited a direct transgranular-type fracture, while Nbss experienced plastic deformation before breaking. Then, tearing occurred along their boundaries.

**Figure 20:**
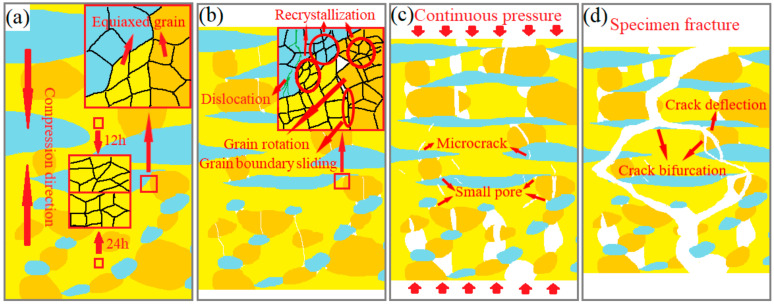
Schematic diagram of fracture feature in the 12–24 alloy during high-temperature compression (pre-compression (**a**), mid-compression (**b**), post-compression (**c**), and final fracture (**d**)). The blue area represents the Nbss phase, the yellow area stands for the Nb_3_Si phase, and the orange area indicates the Nb_5_Si_3_ phase.

According to the above-mentioned analysis, the schematic diagram of fracture features in heterogeneous layered 12–24 alloys during the high-temperature compression process is given in Figure 20. Nbss and silicides displayed distinct deformation and fracture behaviors under uniaxial compression conditions. Plastic deformation concentrated at the ductile Nbss phase under pressure stress resulted in an increase in dislocation density and dislocation strengthening. Coupled with fine crystal reinforcing from a fair amount of fine intermetallic compounds in original heterogeneous layered 12–24 alloys, the preceding elevated-temperature strength (Figure 16) was greatly improved [44]. The grain boundary cohesion became weaker at high temperatures. Therefore, fine equiaxed silicides proceeded with grain rotation and grain boundary sliding more easily, which could produce some pores on the boundary among several grains (Figure 20b). A few small voids also occurred among the fine fragments of silicides, which did not have enough time to complete the recrystallization. Nevertheless, the growth of pores was slowed down under the influence of the dislocation pile-up of the ductile phase and orientation changes after the grain boundary sliding. Subsequently, these pores were also partly filled by deformed Nbss. It was shown to improve strength and plasticity. Subsequently, stress concentration was caused by the different deformation degrees between hard and soft phases. It prompted the microcrack to generate at the junction and expand (Figure 20c). These cracks propagated into the matrix through a transgranular mode, leading to the final material failure.

## 4. Conclusions

New Nb-Si-based alloys with heterogeneous layers containing the same composition (Nb-16Si) were designed and successfully fabricated by layering various ball-milled powders alternately and subsequent SPS. The microstructure of Nb-16Si alloys with HLS exhibits the characteristic non-uniform layers with multiscale and multiphase structures. It is found that the source of cracks influences their subsequent propagation of Nb-16Si alloys with HLS, and as a result, the room-temperature fracture toughness is significantly improved. The fracture features, including crack bridging, crack deflection, crack branching, and the shielding effect, are particularly outstanding when the initial crack is located in the soft layer and extends across the interface to the hard one. Compared with homogeneous alloys, the room fracture toughness of 12→24 (14.89 MPa·mm^1/2^) increased by 33.8%. Furthermore, the non-uniform deformation arises from different deformation and fracture behaviors in each heterogeneous layer. It contributes to the excellent strength and plasticity of the 12–24 alloy at 1250 °C, with the co-activation of fine grain, dislocation multiplication, dynamic recovery, and recrystallization. This study is of practical significance in promoting the research and application of Nb-Si alloys in the field of ultrahigh-temperature structural materials, such as space vehicles. For this purpose, the thickness ratio of soft and hard layers, the compatibility of morphology and the volume fraction between solid solution and intermetallic compounds in separate layers, and the detailed process flow still need further research and explanation.

## Data Availability

The original contributions presented in the study are included in the article, further inquiries can be directed to the corresponding author.
